# Digital Entrepreneurship for the “Decade of Action”: How Entrepreneurs Can Impact Our Race Towards the Sustainable Development Goals

**DOI:** 10.1007/978-3-030-53914-6_15

**Published:** 2020-06-25

**Authors:** Manouchehr Shamsrizi, Adalbert Pakura, Jens Wiechers, Stefanie Pakura, Dominique V. Dauster

**Affiliations:** 1 Hanze University of Applied Sciences, International Business School, Groningen, The Netherlands; 2 Loughborough University, School of Business and Economics, Loughborough, Leicestershire UK; 3 Nordhausen University of Applied Sciences, Chair of Digital Management, Nordhausen, Germany; 4 gamelab.berlin of Humboldt-Universität and RetroBrain R&D GmbH, Hamburg, Germany; 5 RetroBrain R&D GmbH, Hamburg, Germany; 6 Mensa International, Riskful Thinking Ventures LLC, Cologne, Germany; 7 University of Hamburg, Hamburg, Germany; 8 Yunus + You - the YY Foundation, Wiesbaden, Germany

## Abstract

In 2020, the UN launched the “Decade of Action” to achieve the *Sustainable Development Goals* (SDGs) by the year 2030. As the SDGs are interdependent, intersectional and interdisciplinary, so must be their solutions. This chapter argues that the best way to identify, develop, and scale solutions of such quality is (digital) entrepreneurship, building on the principles of open innovation, cutting-edge technologies, and social business. The COVID-19 pandemic in early 2020 in particular serves as a stark reminder of the interconnected nature of the SDGs and the challenges we face in achieving them. In this article, we explore the third SDG (SDG-3), “Good health and well-being”. We show the potential for digital entrepreneurship to foster the rise of new forms of digital health care and to accelerate the digitalization of the healthcare sector. Due to both perceived and real issues of regulatory compliance, user experience, and long investment/equipment use cycles, SDG-3 has been one of the slowest to adopt innovative solutions by far. We discuss specific areas, such as blended reality or quantum computing, for emerging and future digital health applications. In this chapter, we provide: the “memoreBox” of social start-up RetroBrain R&D, a special edition of gamelab.berlin’s app “Singleton”, and D-Wave’s free access to its cloud quantum computing services. All these examples of digital entrepreneurship utilize in whole or in part a combination of *open innovation*, *future and emerging technologies*, and *social business*, thus supporting our rationale. The article closes with recommendations for different stakeholders of entrepreneurial ecosystems, demonstrating both the necessity and the potential of digital entrepreneurship for the SDGs and the “Decade of Action”.

## Introduction

We have a choice—either we go back on the old tracks, or we build new tracks to take us to a new civilization. We are now in position to build new tracks. We missed our chance in 2008 in building those after the global financial crash. Let us not miss the chance this time.

Muhammad Yunus^1^

The 17 Sustainable Development Goals (SDGs) initiated and adopted by all United Nations Member States in 2015 have been a driving force behind numerous initiatives and projects around the world. They constitute an *agenda for sustainable development* that “provides a shared blueprint for peace and prosperity for people and the planet, now and into the future” (United Nations Department of Public Information 2015, p. 1) while also serving as calls to action for a better future. At their core, SDGs are interdisciplinary, intersectional, and interdependent and address a variety of areas that are of critical importance for both humanity and the planet: environmental protection, ending hunger, and reducing inequality are closely linked to, e.g. sustainable consumption and management of natural resources, improving education and providing elementary health care and sanitation for all. Still, five years into the programme timeframe, many initiatives and projects still fail to address this fundamental interconnectedness. These risks fall short of not only their potential, but also interference and competition for already scarce resources. In consequence, the UN declared the 2020s to be the “Decade of Action” (Guterres 2020, p. 1) and has since then appealed to states, corporations, non-governmental organizations, and other stakeholders to more consistently and deliberately combine forces in order to deliver on the goals set out in 2015 (United Nations 2020).

The global COVID-19 pandemic that began to unfold in late 2019, severely shuttering the global economy starting from February 2020 and expected to cause the worst global recession in almost a century (BBC 2020), serves as an additional stark warning of just how necessary an alignment of forces is. At the time of this writing (end of May, 2020), despite rapid and extensive public health measures being taken in many countries, there are more than 5.4 million confirmed cases and over 345.000 deaths (WHO 2020). The outbreak of COVID-19 not only sent whole countries into lockdown, but also demonstrated how relatively ill-prepared the world is for a global health crisis, even one that has long been anticipated: Corona viruses, like influenza viruses, have been the cause of previous pandemics and have been actively studied as likely candidates for future pandemics. Despite drawing on lessons learned from recent pandemics caused by CoV, e.g. SARS, MERS, the global response has been mixed (Park et al. 2020; Malik et al. 2016; Hayward et al. 2014), partly because of inadequate databases, comparable to other global public health challenges like antibiotic-resistant infections (Shamsrizi et al. 2020). In many cases, the responses to the crisis from governments, healthcare professionals, and the public demonstrate a significant gap between the claimed commitment to the ideals of SDG-3, i.e. “Good health and well-being”, and actual reality in the face of a crisis. This is of particular relevance as health (SDG-3) serves as a foundation for many of the other SDGs (Rosling et al. 2018). Considering the current situation, the slow adoption of digital health in general and digital therapeutics in particular—partly because of plausible reasons (including issues of trust, data protection and reimbursement)—over the past couple of years seems alarming. Still, *digital health* and especially *digital therapeutics* are expected to have a tremendous and positive impact on society if they are adopted by more and more patients, doctors, and other healthcare professionals (Deloitte 2019). One way to foster digitalization in the healthcare sector and to bring better care to more people is through digital entrepreneurship. Technological developments and advances in infrastructure create various opportunities for entrepreneurs (Kraus et al. 2018). However, research on digital entrepreneurship is still in its infancy (Kraus et al. 2018).

In this chapter, we apply a holistic perspective and see entrepreneurship as more than just starting up a new business. Following Hsieh and Wu (2018), we understand entrepreneurship as “the process of designing, launching, and running new business” with its distinct characteristic of “new value creation” (Hull et al. 2007). However, entrepreneurial activity arises from the interplay of stakeholders, institutions, and entrepreneurs themselves (Palmer et al. 2018). Referring to Kraus et al. (2018), providing a state-of-the-art literature review of “Digital Entrepreneurship”, we understand digital entrepreneurship “as a “subcategory of entrepreneurship in which some or all of what would be physical in a traditional organization has been digitized” (Hull et al. 2007, p. 293) and is thus defined as “the sale of digital products or services across electronic networks” (Guthrie 2014, p. 115). To summarize, due to the numerous opportunities for entrepreneurial activity, created through digitalization (cf., Hull et al. 2007) and its ability to develop interdisciplinary and intersectoral solutions for complex problems (Breidenbach et al. 2020), digital entrepreneurship offers an impactful instrument for the advancement of sustainable innovations (Kraus et al. 2018), thus the SDGs in general.

## Digital Entrepreneurship as a Game Changer for Sustainable Development Goals (SDGs)

Every new tech-generation makes our societies more inclusive, healthy, and democratic and leads to our institutions having greater transparency and accountability (Pinker 2018). Through digital transformation, which can generally be understood as the “disruptive implications of digital technologies” (Nambisan et al. 2019, p. 1), many new business and science areas have spawned—and numerous implications for culture and society will most likely be enormous (Hausberg et al. 2019). Murphy et al. argue that it is *entrepreneurship* which has been the main driver for the increase in (western) per capita income over the past 200–300 years (Murphy et al. 2006). Entrepreneurship can transform whole industries and scale solutions in a quicker and more agile way than other economic approaches. It is not only one of the “transversal key competences applicable by individuals and groups”, (Bacigalupo et al. 2016, p. 10) as defined by the European Commission, but also a key driver for economic growth “at the heart of national advantage”, as Porter (1990, p. 125) noted. Digital transformation has had an enormous impact on most aspects of daily life and has also changed the way organizations and whole industries operate (OECD 2019), facilitating new types of work and self-employment—and paving the way for digital entrepreneurship: “the enterprising human action in pursuit of the generation of value, through the creation or expansion of economic activity, by identifying and exploiting new ICT [Information and Communications Technology] or ICT-enabled products, processes and corresponding markets” (Bogdanowicz 2015, p. 4). The pervasive accessibility of Internet services has lowered the barriers to start a project, organize, and interact online; this fosters ever-new forms of digital entrepreneurship, especially by allowing even those who could not or would not have formed a company traditionally to find an audience and a market (Allen 2018). At the same time, the current state of accessibility and inclusiveness should not be overstated: it is still the privileged elite that utilizes and benefits from digital entrepreneurship opportunities the most (OECD/European Union 2019). When the United Nations Millennium Development Goals (MDGs) were formulated in the year 2000, digital technology had already become a major part of everyday life, but few foresaw the degree to which it would permeate our lives only fifteen years later. In consequence, where the MDGs were mostly formulated in a technology-agnostic manner, the SDGs embrace the central role digital interconnectedness and technology generally have to play in improving the state of the world (Noville-Ortiz et al. 2018).

New ventures can and, more importantly, have a strong incentive, to catalyze structural changes in sectors currently held by large incumbents, whose incentives usually lie with maintaining the status quo (Apostolopoulos and Liargovas 2018; Hockerts and Wüstenhagen 2010). While it is by no means a given that entrepreneurs will be intrinsically motivated towards founding ventures which particularly take into account the SDGs, recent data from countries such as Germany is encouraging. It shows a trend towards more new ventures directed at solving social challenges, expanding renewable energy or improving health (Bundesverband Deutsche Startups 2018). Start-ups are able to challenge established companies by disrupting “existing conventional production methods, products, market structures and consumption patterns, and replace them with superior environmental and social products and services” (Schaltegger and Wagner 2011, p. 223). If this trend is to be harnessed and further encouraged, it is crucial to understand (a) what motivates these entrepreneurs, (b) whether their ventures actually end up providing a sustained and positive impact towards the transition to a “sustainable and resilient path” as laid out by the United Nations (General Assembly of the United Nations 2015; Apostolopoulos and Liargovas 2018), and (c), if not, what can be done to assist or direct them towards providing such benefit. At present, research into these questions remains scarce (Moon 2018). To conclude, we contend that digital entrepreneurship might have the biggest impact on the SDGs, if it is successful to utilize three concepts: *open innovation*, *future and emerging technologies,* and *social entrepreneurship.* To show how these concepts can help digital entrepreneurs achieve their goals, we will explain each of the three concepts and present examples as case studies of impactful implementations. While every single concept in itself can help elevate digital entrepreneurship in a meaningful way, we argue that a combination of all three may have the biggest impact on the challenges linked with the SDGs, which shall be elaborated using SDG-3.

### Open Innovation as a Key Driver for Digital Entrepreneurship to Enhance SDGs

Open innovation provides a central element in speeding up the digitalization in the healthcare sector through the development and implementation of innovative technologies. As the United Nations Conference on Trade and Development stated (2017), we need “digitally enabled open and collaborative innovation: Fostering open, digital collaborations. Such innovation approaches draw on and recombine multiple sources and forms of knowledge, especially through digitally enabled open collaboration”. However, as von Geibler et al. (2019, p. 20) argue, “this early innovation stage proves to be a challenge for corporate practitioners and innovators, largely due to the concept’s intangible, qualitative nature and the lack of data”.

Open Innovation evolved into an approach that many incumbent firms use regularly. They do not rely solely on knowledge generated within the company, but also facilitate knowledge outside their company to innovate (Bogers and West 2012). Chesbrough (2003) argues that the border between firms and their immediate intellectual environment is not impermeable and therefore enables companies to acquire new knowledge. Sources of valuable knowledge for innovation can be customers, suppliers, and universities (Dahlander and Gann 2010; Brunswicker and Vanhaverbeke 2015). Start-ups face different challenges than incumbent firms, but can just as well facilitate open innovation to succeed. They often lack intangible (e.g. technological expertise) and financial resources (Baum et al. 2000) and are seldom able to form strong strategic alliances (Freeman and Engel 2007). By opening up to external partners (outside in), start-ups are able to compensate for their resource constraints which can positively affect overall firm survival (Eftekhari and Bogers 2015). As Pakura (2020) points out, open innovation acts “as a driver for new organizations”, which is especially true at three levels of impact: *firm development*, *technology development*, and *technology commercialization*. The findings show that start-ups can use different types of relationships with a variety of network partners in order to drive the development and commercialization of innovations. Such relationships can range from loose and informal networking ties to close and formal partnerships, e.g. R&D collaborations with universities and incumbent firms. Although all types of relationships can forward innovation processes of start-ups, Pakura (2019) concludes that “synergetic partnerships, such as R&D collaborations with universities and incumbent firms, create opportunities at all three levels” and that innovation benefits the most from those partnerships. Recent findings suggest that increased links to and knowledge flows from various external partners, particularly in uncertain environments, lead to improved innovation outcomes (West and Bogers 2011). Especially towards the end of the twentieth century, the shift from closed innovation approaches to open innovation models was fuelled by the emergence of digitalization processes (Bogers and West 2012). While the world became more and more digitized, open innovation became a key driver for entrepreneurship and allowed for reducing research costs, spreading risks, and commercializing innovations faster and on a global scale. In recent years, open innovation has been successfully applied in many industry contexts, for example, health care and IT, as well as in academic entrepreneurship (Siegel and Wright 2015), government innovation (Gascó 2017), and social innovation businesses (Nambisan et al. 2019). Chesbrough (2020, p. 3) pointed out how “[o]pening up will speed up [the firms] internal innovation process, and allow you to take advantage of the knowledge of others in your business (outside in), even as you allow others to exploit your knowledge in their business (inside out)”. Opening up has the power to create even more experiments, generate more knowledge, and explore more ways to apply that knowledge for challenges (Chesbrough 2020). It can help solving a variety of challenges, but those with a higher level of complexity profit the most from this interconnected approach. The more complex a challenge seems, the more a firm must engage in extensive knowledge sharing to get closer to a solution. Furthermore, opening saves time, which is critical in the healthcare sector, especially when facing a pandemic (Chesbrough 2020). In a global pandemic, where time is of the essence, openness and open innovation can even save lives (Chesbrough 2020). To conclude, digital entrepreneurs that engage with large-scale problems, and/or want to impact complex ecosystems (like the healthcare sector), must consider open innovation approaches.

### Future and Emerging Technologies as Enablers of Digital Entrepreneurship Towards SDGs

While the future is arguably uncertain and many believe that we are living in an “Age of Paradox” (Handy 1995), there are several future and emerging technologies that entrepreneurs can exploit today or where entrepreneurship can profitably contribute to the development or implementation of future technologies. Thinking ahead and implementing future technologies can give entrepreneurs a competitive edge or even enable them to create entirely new markets. So-called future and emerging technologies (FETs) are also part of the “Horizon 2020” programme by the European Union with the goal to “create a fertile ground for responsible and dynamic multidisciplinary collaborations on future technologies and for kick-starting new European research and innovation ecosystems” (Horizon 2020, 2018, p. 4). Future and emerging technologies are self-evidently complex and not widely known and implemented. Implementing them requires a strong strategic focus and the ability to innovate by means of tools that are currently not available in the mass market. Moreover, deeper factors are necessary to obtain economic and social value from technology. Generating technology alone is insufficient and must also be broadly disseminated, and then absorbed and put to work before its full value could be realized, as Chesbrough (2019) argues. To get a short overview of presumably impactful FETs, the World Economic Forum (2020) created an overview that we adopted (Table 1) and that shows not only how FETs like artificial intelligence and quantum technologies will potentially shape our future, but also how they will affect the different SDGs.

**Table 1:** Examples of future technologies

Technology	Impact on the following SDGs	Technology	Impact on the following SDGs
Quantum computing determined optimal carbon capture material	SDG-7, SDG-13	Ultra-high speed, zero-emissions long haul transport, including underground, surface, aviation, shipping and drones	SDG-7, SDG-9, SDG-11, SDG-13
4IR-enabled deployable nuclear fusion using AI to predict disruptions that halt feasibility	SDG-7, SDG-13	Zero-waste advanced materials for clean energy and advanced waste heat capture and conversion	SDG-7, SDG-9, SDG-11, SDG-12, SDG-13
Advanced materials for generation of low-cost and zero-emissions gaseous fuels, incl. ammonia and hydrogen	SDG-7, SDG-13, SDG-14	Quantum-enabled extreme efficiency data centres and supercomputers	SDG-7, SDG-9, SDG-12, SDG-13
Genetic rescue and genome modification for endangered and extinct species and resilience	SDG-14, SDG-15	4IR-enabled internet connectivity for all (drones, satellites)	SDG-1, SDG-4, SDG-5, SDG-8, SDG-9, SDG-10, SDG-11
Attracting and removing micropollutants (synthetic biology)	SDG-6, SDG-11, SDG-13, SDG-14, SDG-15	Quantum cryptography for the prevention of cyberattacks on AI/quantum computers	SDG-9, SDG-16
Low-zero emissions and ultralow-cost desalination technology using advanced materials	SDG-3, SDG-6, SDG-13	AI-enabled privacy-protected, public good digital health platform collating healthcare data, sensors, wearables and genomic data	SDG-3, SDG-16
End-to-end automated, connected and optimized food and fibre system, incl. elimination of spoilage, loss and waste	SDG-2, SDG-12, SDG-13, SDG-15	AI-enabled development of new antibiotics to address microbial resistance to current antibiotics	SDG-3, SDG-10
Low-cost, low-GHG emissions synthetic proteins (AI and synthetic biology)	SDG-11, SDG-12, SDG-13, SDG-15	4IR-enabled “access to care” digital technologies, distribution and delivery systems	SDG-3, SDG-10
Advanced materials for durability of energy-intensive products and materials	SDG-2, SDG-9, SDG-12, SDG-13	Decoding well-being and longevity using AI and sensors for personalized health maps and sequenced genomes and phenotypic data	SDG-3, SDG-10
Zero-emissions chemicals, steel, aluminium, cement using advanced materials and/or biotech (e.g. biocement)	SDG-11, SDG-12, SDG-13	Gene editing (e.g. CRISPR) to tackle human diseases driven by gene mutation	SDG-3

*Source* Adopted from: World Economic Forum (2020)

While we cannot go into detail regarding the different technologies and their respective effects on society, we will focus on two major technological concepts that we assume will have tremendous impact on achieving the SDGs and which we will take up and reflect in our case studies (see Chap. 10.1007/978-3-030-53914-6_4): *Quantum Computing* and *Blended Reality*.

Although it might sound puzzling, quantum technologies are already widespread: “computers, data networks and the majority of medical imaging techniques could not have been achieved without quantum effects. This is because components such as transistors, diodes and lasers all make use of principles of quantum physics” (Federal Ministry of Education and Research 2018, p. 6). These are examples of first-generation quantum technologies that started as scientific endeavours which were then implemented in a myriad of ICTs and everyday devices that we use today. Almost a century after the field of quantum physics was created in Central Europe, an increased understanding of those quantum technologies is now creating new opportunities. As Krutzik and Shamsrizi (2020) outline, the “second quantum revolution” will massively impact the twenty-first century, and is widely seen as “[that which] comes *after* the digital transformation”. The manifold areas in which this impact can be seen include “measuring devices with much higher precision, vastly enhanced data communication security, and […] higher-performance satellites and computers” (Federal Ministry of Education and Research 2018, p. 6). Quantum technologies and their specific applications are based on quantum principles that, in turn, exploit the unique physical principles of the quantum world.

**Fig. 1:**
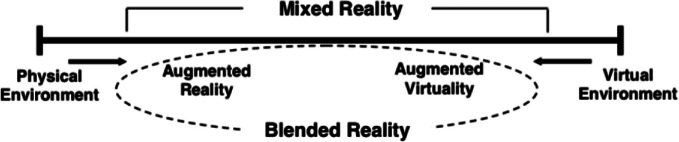
Blended reality in relation to the physical-virtual environment continuum. *Source* adapted from Milgram and Kishino (1994), in Bower et al. (2010)

The second example of a potentially impactful FET is the concept of so-called blended reality: Many Health and Exergames use virtual or augmented reality to promote active living and exercise despite the still widely held preconception of gaming being an “unhealthy” (or at least not health-positive) activity. The popular VR rhythm-game *Beat Saber,* for example, is “widely considered a good option for exercise in VR” and uses the technology to reach people at home and motivate them to move and stay healthy (Fingas 2020). In a study on the potential health impact of Pokémon Go, Duke University’s School of Medicine was able to show that “increases in physical activity were highest among individuals who stood most to benefit from additional activity, such as individuals who are overweight or obese, or who get little regular exercise to begin with” (Will Will 2017). Another illustrative example is provided by blended reality exercise equipment or applications, such as those provided by Peloton (onepeloton.com). Their smart exercise equipment enables its users to sign up for training regimes overseen by remote trainers, to exercise and receive instruction “together” via integrated video conferencing. Other offerings such as *Supernatural* even allow for exercise in full virtual reality (Oculus 2020). Many of these technologies are actively used today, but big technological leaps will make true “Blended Realities” a part of our everyday life. Steincke defines blended reality as the seamless transition between the fully physical and fully virtual, described as a continuum between these two poles. Steinicke (2016) anticipates that in about 30 years, virtual and “real” reality will not only be blended, but even merged, and humans will not be able to perceive any difference The consequences of such a situation have been described as potentially even turning “real” reality into a “homeopathicum” (Sedláček and Shamsrizi 2017) (Fig. 1).

### Social Business as an Essential Element Towards SDGs

**Fig. 2:**
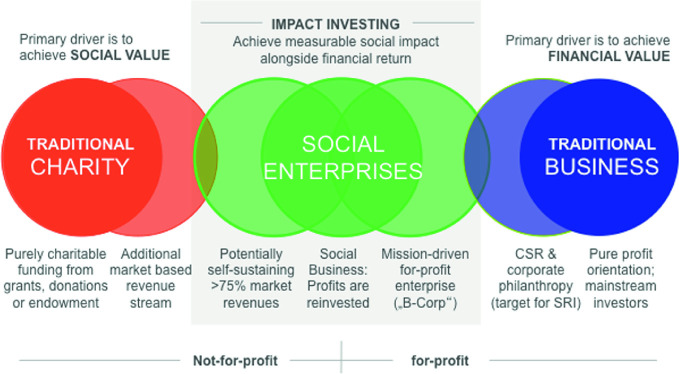
Continuum of varieties of organisations in the “new capitalism”. *Source* Ryder and Vogeley (2018)

As we are entering the second decade of the new millennium, one can observe rather unexpected changes even among thought-leaders of both theory and practice in economy and business: Michael Porter wants his students to create *Shared Value* (Porter and Kramer 2011), BlackRock is “making sustainability integral to portfolio construction and risk management” (Fink 2020) and lets its portfolio companies know that “purpose is the engine of long-term profitability”, and the founder of the World Economic Forum, Klaus Schwab, opened this year’s WEF Annual Meeting by pointing out that while “‘stakeholder capitalism’ has been around for a half-century, it has only recently begun to gain traction against the prevailing shareholder-primacy model of profit maximization” (Schwab 2019). Consistently, the “Ethics in Action”-initiative of the UN Sustainable Development Solutions Network pointed out that “the challenges of sustainable development are primarily ethical in nature”; thus, “the Sustainable Development Goals require ‘moral capacity’ as much as financial or technical capacity” (Annett et al. 2017). At the core of this SDG-driven transformation is the idea of a “new capitalism”, in which both traditional for-profit (blue, cf., Fig. 2) and not-for-profit (red, cf. Fig. 2) organizations are complemented by social entrepreneurial actors in all of their varieties (green, cf. Fig. 2), including the supporting impact investing ecosystem surrounding them:

While “debates about the definition of social business versus social entrepreneurship keep coming up at conferences”, the scientific community is “getting closer to clearer definitions” (Grove, as cited in YY Foundation 2019, p. 22). Independently of the definition, it seems that social entrepreneurs may play a major role in creating more inclusive societies (European Commission 2015) and solving the most pressing issues of our time. In particular, social businesses “work in many different areas where they often have a direct impact, such as health, education and infrastructure”, as Gass sums up (Gass, as cited in YY Foundation 2019, p. 30). Regarding the definition of social business, the OECD (2014, p. 188), for example, follows a twofold definition of Muhammad Yunus: Type 1) a “‘non-loss, non-dividend company’ that creates social benefits through the nature of its products, services and/or operating systems”, and Type 2) a “profit-maximizing company owned by its poor or otherwise disadvantaged target beneficiaries, or by a dedicated trust”. As such, the concept of social businesses is notably distinct from any form of charity. In this sense, combining digital entrepreneurship and social business, we assume that stakeholders are enabled to create scalable solutions—especially in the light of the “Decade of Action”. Furthermore, it has also been argued that large corporations/multinational enterprises (MNEs) “[need] a change of course to achieve the UN’s Sustainable Development Goals by 2030” (Bruysten et al. 2020). This transformation is strongly driven by “a breed of entrepreneurs who work as employees within companies to develop business solutions for social or environmental problems:” social intrapreneurs. The OECD anticipates that “social businesses can create new sources of income, raise productivity, reduce ‘aid’ dependency and provide low-income consumers with access to products and services for their basic needs” (OECD 2014, p. 187). With the pressing issues in front of us and the COVID-19 pandemic as a huge “call to immediate action”, solutions that tackle a SDG like “Good health and well-being” should and can facilitate both of these worlds, as “Social businesses will have a direct impact on whichever SDGs they engage in” (Gass, as cited in YY Foundation 2019, p. 30).

## A Conceptual Framework and Canvas of Digital Entrepreneurship for a “Decade of Action”

We see digital entrepreneurship as a necessary component in achieving many, if not all, of the SDGs. A variety of conceptual models, policy frameworks and measurement instruments have been developed to study the driving and impending factors influencing digital entrepreneurship as well as the factors influencing organizational decision-making which furthers sustainable and more generally SDG-oriented business practices. Many of these frameworks, however, adopt a macro-perspective with a focus on the incentives and obstacles faced by multinational enterprises, or organizations that are designed to quickly scale to a global level (George and Bock 2011; George et al. 2016). Yet the vast majority of all enterprises in both highly industrialized and less developed countries are actually small- and medium-sized enterprises (SMEs) (Ayyagari et al. 2017; European Union 2018; Small Business Profile 2018). While the disproportionate impact of MNEs on the overall sustainability should not be understated, SDG-oriented Digital Entrepreneurship, presenting the right overall conditions, potentially may rapidly develop and adapt to niche opportunities. This is due to the domain expertise of its founders and significantly lower regulatory, organizational, and structural constraints with the SDGs being nevertheless supported through socially/environmentally responsible practices. At the same time, it seems unlikely that any single framework could adequately quantify and qualify the wide variety of factors that influence the entrepreneurial activities of SMEs. Following the argument put forward by Kuratko et al. (2015), we agree that only a synthesis of multiple frameworks has any potential to adequately represent Digital Entrepreneurship, especially social digital entrepreneurship. All economic systems are complex networks that are interconnected and interdependent (Bair and Palpacuer 2015; Rasche et al. 2013), and the formation of networks among entrepreneurs, the start-ups they create, and the SMEs they become have been found to be crucial to success (Austin et al. 2006; Dacin et al. 2011). Based on these underlying considerations, we explored the possibilities to help potential digital entrepreneurs to successfully support the SDGs thus positively impacting the “Decade of Action” through the structured application of open innovation, social digital business approaches, and future and emerging technologies. To use these concepts effectively, we developed a special variant of Osterwalder’s Business Model Canvas (BMC) (Osterwalder and Pigneur 2010). Our “*Digital Entrepreneurship for the Decade of Action*”—Canvas (short: “Decade of Action”-Canvas) adds multiple layers to the well-known version by Osterwalder to let digital entrepreneurs better engage with the SDGs.

The canvas implements three major new aspects, which we derive from our theoretical triad of open innovation, future and emerging technologies and social (digital) business. These new aspects will directly help future digital entrepreneurs to evaluate how their solutions benefit the SDGs. First, in this canvas, not only the “usual” value propositions are to be explored, but, referring to the definition of social business, also the proposed value to the SDGs. This means that the potential project and its value proposition needs to relate to the SDGs and to explain how it supports achieving them. Second, we refer to the concept of open innovation and the importance of multiple and different types of relationships with a variety of network partners in order to drive the development and commercialization of innovations. We delimited key environmental actors and influencers from key partners. By answering the question “Who is mostly impacting your field of impact/SDGs in the next ten years?” potential entrepreneurs learn that it is often the network to regulatory authorities or other societal or economic multipliers that can bring a competitive advantage. Working on your network and keeping key actors that affect your field of impact can pay off early on. Third, Beneficiaries are of utmost importance to consider: in contrast to customer segments, thinking about beneficiaries enables digital entrepreneurs to embrace the “triple bottom line”, where environmental, social, and governmental actors benefit. This sensitization is supported by referencing concepts like Ashoka’s Theory of Change, or the social business approach (Drayton 2003). Fourth, referring to future and emerging technologies, “Key Activities” and “Key Resources” force the digital entrepreneurs to re-evaluate their solutions with regards to other, more emerging technologies, which might have the potential to improve the impact and/or efficacy of their approach.

**Fig. 3:**
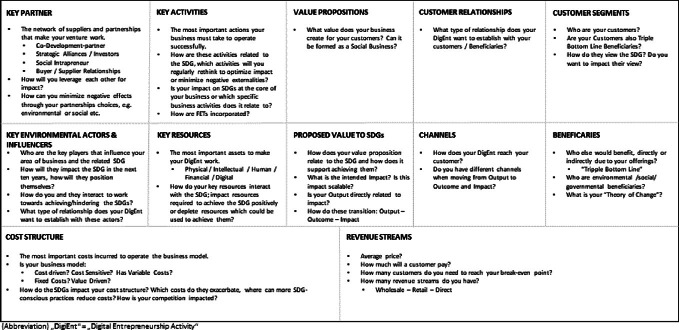
“Decade of action”—Canvas. *Source* Own table (adapted from Osterwalder and Pigneur 2010)

To summarize, we developed the “Decade of Action”-Canvas with these four specific adjustments, whereas the other fields of Osterwalder’s BMC remain mostly unchanged (Fig. 3).

The most current version can always be found at http://www.doacanvas.org/.

## Case Studies

### RetroBrain R&D GmbH: MemoreBox

Germany’s Federal Ministry of Health’s Health Innovation Hub responded, among others, to the COVID-19 pandemic by compiling a list of recommendable “Digital Tools”, which either mitigate COVID-19 directly or help address its wider societal impact (Health Innovation Hub 2020). One of the companies mentioned on this list is **digital-therapeutics** company RetroBrain R&D, a spin-off of Humboldt-Universität’s Cluster of Excellence. RetroBrain R&D develops a fully gesture-controlled video game console named “memoreBox”, which has been called “a benchmark in the therapeutic gamification industry” (LIFT Basel 2015). The overall goal of RetroBrain’s solution is to extend the quality of life of the elderly by developing state-of-the-art, evidence-based therapeutic video games. The video game system—classified as a class 1 medical device—supports the prevention of typical age-related diseases and accompanies the therapy of diseases such as dementia or Parkinson’s disease.

In a pilot project under the patronage of among others Germany’s Minister of State for Digitalization, which studied the health-promoting effects regular gaming has on the social, physical, and cognitive resources of senior citizens, the findings were clear: Compared to non-gamers, gamers showed significant improvements in cognitive performance, gait stability, motor skills, stamina, and coordination. There are also moderate improvements as it pertains to the health-related quality of life, the extent of which is practically significant. There were also positive trends in the subjective experience of pain, which was reduced by regular gaming. As a result of this study, “BARMER [one of Germany’s largest health insurance funds] is convinced of RetroBrain’s memoreBox”, as Dr. med. Mani Rafii, member of the board, comments: “The concept combines movement with enjoyment and games and makes it possible for elderly people to remain mentally and physically fit and to actively participate in society. Since we had positive experiences with the concept within the framework of a pilot phase, we are now rolling it out nationwide, so we can give even more elderly people the opportunity to take part in the preventive and health-promoting capabilities of this video game platform” (Rafii, M., as cited by Jakob-Pannier 2019, p. 1). In 2019, Germany’s National Association of Statutory Health Insurance Funds commissioned the Institute for Innovation and Technology of VDI/VDE-IT to conduct a study on the potential impact of digital tools in care and nursing. According to this study, the memoreBox “proves, how people in need of care profit from the use of a digital tool in different fields including their cognitive abilities, social interaction and conclusion, as well as gait quality”, and furthermore even the nursing staff benefits” (GKV-Spitzenverband 2019, p. 151). What opened memoreBox the door to the Healthcare Market was the German Act to Strengthen Health Promotion and Preventive Health Care, which has been in effect since the summer of 2015. The need for this law shows how diametrically opposed the two poles of “having fun” and “getting/being healthy” were at that time. The legislator created this law to motivate the health insurance industry to invest more money in prevention. Given that it generally takes fewer resources and is more promising to keep people healthy—instead of trying to heal them after they have taken ill, which takes much more effort and has far lower chances of success,—the legislator created the Prevention Act obligating health insurance companies to allocate sufficient funds to promote meaningful prevention. Like many social business start-ups making use of digital technology, RetroBrain R&D operates in an ecosystem of cross-sectoral quality; besides the “PEP Program” of Ashoka, one may particularly mention the “Impact Factory”.^2^ Thus, RetroBrain R&D can be studied as an exemplary case for aspects like “key partner”, “key environmental actors and influencers”, and “beneficiaries” of our “Decade of Action”—Canvas.

### gamelab.Berlin: Singleton and the #WeVsVirus-Hackathon

Suggesting that “we as a society [can] work together to master the challenges that arise in the wake of the Corona (COVID-19) crisis with new solutions”, the Federal Government of Germany had invited people to take part in a virtual “#WeVsVirus” hackathon (https://wirvsvirushackathon.org). Under the patronage of the head of the Federal Chancellery and supported by the Federal Government’s “Digital Council”, more than 40,000 participants developed digital solutions to problems related to the COVID-19 pandemic. These participants spent 48 h working on altogether 1500 ideas, of which many can be classified as digital entrepreneurship and/or social businesses. Overall, “the model for this attempt to find digital solutions in the global fight against the coronavirus pandemic was a similar event in Estonia” (Hänel 2020, p. 1) and was considered as an exceptional and sophisticated approach of open innovation by a public administration (Gegenhuber et al. 2020). The issues dealt with were mostly the following: 1. Spread of Sars-CoV-2, 2. Provision of Medical Care, 3. Politics Administration, 4. Economy, 5. Solidarity (including Education), 6. Living in (Self-)Isolation; evidently, these issues span all SDGs. As we focus on SDG-3 in this article, we will exemplarily describe the project “Singleton #WirBleibenZuhause”, which was chosen to be included in the “Solution Enabler Program”—by which the German government wants to implement the most promising solutions of the hackathon—and which is an especially promising digital solution for mental health in (Self-)Isolation. Originally a research project at gamelab.berlin, Singleton is now offered by the social business spin-off Homo Ludens GmbH, making this case a prototypical example of digital entrepreneurship as a vehicle for social impact.

Singleton is a card game that gamifies time management while encouraging mindfulness in order to help those who traditionally struggle to adhere to to-do lists and tasking. It was initially designed as a physical game and played by the inventor and his fellow researchers at gamelab.berlin (Lilge and Stein 2018). A digital version for Android and iOS was eventually created and open sourced, making it available to a wide community of developers. 60 Singleton cards with entertaining and socially activating challenges were created, which together formed a game that was highly adapted to the organizations needs. Singleton began to be used in all kinds of organizations and companies as a new way to deal with change. In the course of focusing on organizations, the scientists of gamelab.berlin spun out of the university and created a company with the goal of bringing the cultural technology of the game to areas of society that are typically not interested in games. The aim of the spin-off was to design systemic and individual learning processes in such a way that the most natural of all forms of learning motivation could be activated: the joy of discovering something new. In the context of university research, it had already become clear that games can do much more than just entertain. Based on the principles of open innovation, Gamelab developed games for data collection in research, political education, school education on climate change, and neurosurgical training and even created a game for people suffering from lethal diseases. It turned out that game mechanics and forms of motivation have an effect far beyond what we are used to calling games—into the most serious areas of society. The ideas still come from research and the connection with their scientific work remains, but being a social business allows them to have faster development cycles and opportunities for practical applications, which have a concrete impact on the SDGs through Digital Entrepreneurship. The new version of the game enables people to play it for themselves and at home, learning to be more mindful and improve their well-being and health, thus directly helping achieve SDG-3. In this case, expanding the development of Singleton in the course of the #WeVsVirus hackathon is a useful example of how to align the aspects “proposed value to SDGs”, “key partner”, “customer relationships”/“customer segments”, and “beneficiaries” of our “Decade of Action”—Canvas.

### D-Wave Systems: Access to Quantum Computer Processing for Projects Addressing COVID-19

As we have indicated above, quantum computing will certainly have wide-ranging implications and a substantive impact on all aspects of life in the future. This obviously includes the SDGs. Witold W. Kowalczyk of Harvard-spinoff Zapata Computing (2020), a quantum computing software company, identifies five SDGs in particular that will be impacted by the novel computing resources quantum computing provides:

- Zero hunger (SDG-2) via new algorithms for crucial soil composition analysis, nitrogen fixation, etc.
- Good health and well-being (SDG-3) via increased velocity of drug discovery and simulation.
- Clean water and sanitization (SDG-6) via optimized water distribution, new catalyst discovery/development
- Affordable/Clean energy (SDG-7) via advances in materials science leading to, e.g. better batteries
- Climate action (SDG-13) through improved meteorological modelling and analysis.

One company that has stepped up in the wake of the COVID-19 outbreak is the Canadian quantum computing company D-Wave, one of the earliest (Lardinois 2019, p. 1) of the latest generation of quantum computing start-ups. They announced that they would support anyone “focusing on new drugs”, but also that they are “open to any research or team working on any aspect of how to solve the current [COVID-19-]crisis, be that logistics, modelling the spread of the virus or working on novel diagnostics”. In addition, their partners^3^ will provide “engineering expertise to teams that are using Leap 2 for developing solutions” (Lardinois 2019, p. 1). Founded in 1999, D-Wave is a privately held company. Quantum technologies are believed to be “driving forward a technological revolution” and to become “the engine of innovations in science, economics and society in the twenty-first century”, as experimental physicist Prof. Dr. Rainer Blatt sums up the results of the 2016 Lindau Nobel Laureate Meeting on the “second quantum revolution” (von der Stein 2016, p. 1). Regarding the COVID-19 crisis as well as the issues targeted by SDG-3 in general, particularly quantum computing is at the core of D-Wave’s impact. According to one of D-Wave’s partners, Prof. Dr. Kristel Michielsen from the Jülich Supercomputing Centre, it is promising “to accelerate the solution of complex problems in pharmacology and epidemiology, such as those that have arisen in the unprecedented COVID-19 crisis, by means of hybrid workflows from quantum-classical computer simulations” (Forschungszentrum Jülich 2020). As Analytics Insight sums up, “The company’s hybrid quantum-classical cloud service could conceivably help researchers simulate molecular interactions between coronavirus and its target cells, or simulate the spread of the COVID-19 disease in complex settings. It could also help planners optimize supply chains and hospital logistics” (Srivastava 2020) In an interview with IEEE Spectrum, gathering the first week’s worth of submissions from coronavirus researchers applying for D-Wave time, the CEO of D-Wave claimed that the initial response to their offer came from teams tackling a range of coronavirus-related problems: “We’ve seen problems being explored in the following areas: (1) the modelling and simulation of the spread of the virus, (2) the scheduling of nurses and other hospital resources, (3) assessing the rate of virus mutation, and (4) the assessment of existing drugs as potential treatments” (Anderson 2020). Opening up access to their resources also allows other companies in the space which are already committed to the SDGs such as ZAPATA, to work with D-Wave and interested parties to leverage domain, quantum-software and hardware expertise, thus showing the potential of the simultaneous consideration of the aspects “proposed value to SDGs”, “key partner”, “beneficiaries”, and “key activities” (notably its sub-aspect “How are FETs incorporated?”) of our “Decade of Action”—Canvas.

## Conclusion and Implications: Making Open Innovation, Social Business, and Future and Emerging Technologies Work for Digital Entrepreneurship and the “Decade of Action”

The current COVID-19 pandemic shows how important the fight for the Sustainable Development Goals really is and how COVID-19 has prompted a wide variety of open, collaborative responses (Chesbrough 2020). While all SDGs are impacted by the pandemic—especially the neglected issues in SDG-3 (good health and well-being) become apparent—and the need for more and better digital health applications became obvious. Digital entrepreneurs now have to step up and build the next wave of impactful start-ups, notably social (digital) businesses, for the Decade of Action. Based on our literature review and practical case studies, we developed the following six primary recommendations for action which target all stakeholder of the digital entrepreneurial ecosystem. In doing so, we provide a holistic lens combining the findings of open innovation, social business, and future technologies:

1. Foster knowledge and technology transfer via open innovation from the scientific community beyond businesses towards all actors working on the SDGs, particularly taking into account the necessary access to finance
2. Foster entrepreneurship education, and expand its scope towards continuing education also focusing on senior and mid-career executives
3. Introduce and support social business (and/or social intrapreneurship) in the potentially impactful organizations working on the SDGs
4. Harness the potential of diversity, notably female entrepreneurship (Halberstadt et al. 2018), migrant entrepreneurship (Council of Europe 2019), introverts (Castrillon 2019), entrepreneurs of colour (Kauffman Foundation 2016), and other forms of minority entrepreneurship (Bates 2012)
5. Include founders and entrepreneurial ecosystems as part of the regulatory and economic policy framework to cope with COVID-19
6. To obtain economic and social value from emerging technologies it is not enough generating technology. Moreover, it is necessary that the technology will be disseminated, absorbed and put to action before its full value can be derived (Chesbrough 2019).

Furthermore, our findings culminated in an easy-to-use canvas. We took Osterwalder’s “Business Model Canvas” and redesigned it to help digital entrepreneurs effectively tackle the SDGs: *open innovation* for complex problems, *future and emerging technologies* for future proof solutions and *social business thinking* to keep societal issues in mind. The relevance of digital health and therapeutics for achieving the SDGs is rising and an event severely restricting access to healthcare professionals and doctors due to capacity overload or isolation, such as the COVID-19 pandemic, serves as a stark reminder of just how fragile many of our achievements towards particularly SDG-3 can be in the face of global calamity. Fostering an entrepreneurial spirit among young people, but especially those who are not traditional founders such as women, the elderly, people with disabilities, refugees, and others while also encouraging open innovation and cooperation within sectors will help build a more inclusive and resilient economy and health sector.

But how can one use the “Digital Entrepreneurship for the ‘Decade of Action’—Canvas” best? Innovative teaching and learning formats at universities and other forms of higher learning—notably those engaged in lifelong learning—have increased, ever since the first formal entrepreneurship education formats were created in the early 2000s. However, many entrepreneurship education curricula continue to disregard the idea of fully Digital Entrepreneurship, not to mention how little is on offer addressing health challenges in particular. As “really big opportunities arise only when brilliant innovation meets overwhelming market needs at just the right time” (von Windheim 2014, p. 35). There are unique opportunities for entrepreneurship education to help shape the digital landscape in Germany and beyond, fostering connections between all stakeholders, which is why—in the spirit of our “Digital Entrepreneurship for the ‘Decade of Action’—Canvas”—universities need to bring together all stakeholder and players potentially involved in founding new ventures (von Windheim 2014). Teaching about the SDGs in the same manner as we more generally teach about business ethics and philosophy, must become the backdrop of our entrepreneurship education if we want to achieve the still ambitious agenda set out by the United Nations and transform our society for the better.

## Footnotes

1 Corona Pandemic: Time Is Running Out Fast, A Letter from Prof. Muhammad Yunus (2020).

2 A joint initiative of a diverse group of founding partners including German family equity company Franz Haniel & Cie. GmbH, Beisheim Foundation, KfW foundation (of the KfW, the German government-owned development bank), and Anthropia gGmbH, a social business itself.

3 i.a. Volkswagen, DENSO, Jülich Supercomputing Centre.
